# Importance of Desensitization for Autistic Children in Dental Practice

**DOI:** 10.3390/children10050796

**Published:** 2023-04-28

**Authors:** Eva Martínez Pérez, Alberto Adanero Velasco, Víctor Gómez Clemente, Mónica Miegimolle Herrero, Paloma Planells Del Pozo

**Affiliations:** 1 Clinical Specialties Department, Faculty of Dentistry, Complutense University of Madrid, 28040 Madrid, Spain; 2 Clinical Dentistry Department, Biomedical and Sciences Faculty, European University of Madrid, 28670 Madrid, Spain; 3 Department of Orthodontic, University Francisco de Vitoria, 28223 Madrid, Spain

**Keywords:** autism, behavior management, child, dental, desensitization

## Abstract

Objectives: Dental treatment in special needs patients, including children with autism, can be accomplished by reducing the behaviors that can reduce fear, as it has been demonstrated in other studies. The present study aims to examine the influence of the latency time elapsing between desensitization and the real dental situation on facilitating the access of children with autism to dental treatment. Study design: Nineteen patients with autism, who were aged 3–14 years and attended the Special Education Center in Madrid but were living with their parents at home, were selected for the study. All children in the sample were subjected to a desensitization process before attending the real dental office. Two study groups were established: the latency period between the last desensitization and the real situation was one day for the first group and seven days for the second group. An experimental study was conducted to assess the child’s cooperation in the dental chair; the dental examination was divided into several steps and the highest step reached by each child was recorded. Results: There is a statistical difference in the number of steps reached between the children who received the information just before the examination date and the children who experienced a longer latency period between receiving the information and experiencing the examination. Conclusions: We would like to emphasize the importance of providing information in advance when dealing with autistic children; this information should be as close as possible to the real situation. Additionally, we would like to stress the importance of inter-cooperation between parents, educators, and pediatric dentists in order to guarantee adequate oro-dental care for autistic children. Further studies with larger sample sizes and a control group are recommended.

## 1. Introduction

Autism spectrum disorders (ASD) are conditions characterized by impaired autonomy and all kinds of social skills. The fifth edition of the Diagnostic and Statistical Manual of Mental Disorders (DSM-5) [1], states that some of the main characteristics of ASD include social communication and deficits, and a lack of interaction, in addition to behaviors that are repetitive. When you compare this edition with the previous edition (DSM-IV-Text Revision [2]), the DSM-5 made important changes. When the authors write about the diagnostic area, the language abilities were de-emphasized. Furthermore, some of the subcategories in the diagnostic area were also eliminated, such as childhood disintegrative disorder, Asperger’s syndrome, pervasive developmental disorders and Rett syndrome. Furthermore, the diagnostic subcategories, that is, autistic disorders and those not otherwise specified, were abolished. The DSM-5 states different levels of severity in the ASD, that go from requiring support, this one being the lowest, to requiring substantial support, to requiring a lot of substantial support [1].

Some studies have addressed the importance of identification as soon as possible for those with ASD, since early treatment is correlated with good outcomes in the long-term when it is intensive. There are many theories about how the patients should be treated and what is the etiology of the syndrome, but there is no consensus yet, and that leaves the families with confusion about how to deal with this illness. It is essential that primary care clinicians have up-to-date information about the science of autism, due to the important role they play in this illness, not only in the early identification but also with the management of the patient [3].

The diagnosis criteria used in the last decades for the ASD led to an increase in the prevalence of these disorders [4].

The changes made in the DSM-5 classification criteria will surely lead to different groups of children having an ASD diagnosis [4,5].

Documented research about the dental condition of children with autism spectrum disorders is limited. However, given the unique habits, such as movement repetition and difficulties when introduced to new things and experiences, of these children detailed by authors such as Tesini and Fenton [6], it is probable that the majority of them require special dental care. Their eating habits and difficulty in learning oral hygiene techniques predispose autistic children to dental complications such as caries and poor oral hygiene [6,7,8,9,10,11,12,13,14]. It is also important to note that self-injurious habits are part of the behavioral disorders of many autistic children [7,8,9,10,11,12,13,14,15]. Regarding the face and mouth, we may see auto-extractions, ulcerations, wounds, and tongue, cheek, and lip bites [15,16,17].

It has also been well documented that children with ASD experience various barriers to optimal dental and oral care [18]; many children with autism exhibit fear that is usually because of the difficulty they have in dealing with novel visual and auditory stimuli, which leads them to anxiety. When this happens, parents choose not to take their children with autism for routine examinations because of the children’s fear and their behavior in new spaces, to protect them from difficult behavioural situations. When we are talking about oral healthcare, parents prefer other treatments such as conscious sedation or general anesthesia [18,19,20].

On the other hand, dental professionals caring for patients with an ASD diagnosis will need to give treatments based on approaches that are more family-centered, giving parents support and understanding in their concerns [21].

The application of behavioral methods to control dental fear in children is always time consuming, especially when dealing with children with autism. Additionally, there are no procedures that are validated for behavioral treatments in these patients, so there is no consensus in the dental community. However, in children with autism, dental treatments can be accomplished using methods commonly known to reduce phobic behavior in other children, such as systematic desensitization and distraction, as demonstrated by Luscre and Center in their study [9].

Different behavioral dental treatment packages have been used to decrease fearful reactions to dental stimuli or increase compliance to dental exams. These procedures include reinforcement, shaping and fading [22]; desensitization, video modeling and reinforcement [19,20,21,22,23]; visual pedagogy [24,25]; and desensitization [26,27,28].

The aim of this study was to determine the influence of the variable “latency time” elapsing between desensitization procedures and the children’s exposure to the real clinical examination on the effect of desensitization.

With this aim, the Association for Investigation and Study of Mental Retardation and teachers of the Department of Prophylaxis, Pedodontics and Orthodontics in the Faculty of Dentistry at the Complutense University of Madrid (Spain) participated in this study focusing on dental treatment in autistic children, and this study was made in one academic year. This study was financially supported by the Ministry of Education and Science of the Autonomous Community of Madrid, Spain.

## 2. Materials and Methods

### 2.1. Sample

The inclusion criteria were children fulfilling the diagnostic criteria of autism according to the DSM-IV, aged between 3 and 14, attending an education center for children with special needs in Madrid, and whose parents or guardians signed informed consent.

The exclusion criteria were any associated syndromes or disabilities (visual, auditive, or motor).

The sample size was calculated using the number of children that attended the Special Education Center, CEPRI. With all the students between the ages of 3 and 14 from the center, a confidence level of 95% and an error margin of 8%, we had a cohort of 19 patients. All participants met the inclusion criteria and were randomly allocated and divided into two groups using a random number generator.

The recruitment was carried out by the educators of the center over the course of 12 months, and after that, the participants presented at the University. The two groups contained 10 and 9 children, respectively.

### 2.2. Methods

#### Information Exchange

First, information was exchanged between the psychologists and pedodontists in the research team by means of lectures and bibliographies. The psychologists gave the pedodontists information about the systematic desensitization they used in the special needs center, and the pedodontists gave information about the clinic, the time required and the methods used during the dental procedures.

The psychologists visited the Department of Dentistry to gather the information that they would pass on to the participating children in advance and to collect any information they might need themselves. They also investigated the physical characteristics of the place where the children were going to be examined, looking for stimuli that could be negative for the participants, such as strong noises and bright lights, in order to avoid them if possible. They additionally took some dental materials for olfactory and taste stimuli, such as topical anesthesia and dental cements used in pediatric dentistry.

Likewise, the dentists on the team visited the educational center that the children attended daily in order to meet them and to help them become familiar with the techniques and protocols. There were several visits during the duration of the project, and there were always at least two dentists at the visit. The aim of visiting the children in the special needs center was for them to become familiar with the presence of the dentist.

### 2.3. Questionnaire

A questionnaire was made with all the information (Supplementary File S1). A pilot study was made in order to validate it. It was distributed to the children’s families after a presentation to the parents by means of a lecture dealing with the most important aspects of oral health in children and after the parents agreed to participate in the project by means of informed consent. Its aim was to gather information about demographic data, clinical data, habits, previous dental experiences (if applicable) and other data related to the child’s behavior in dental offices, among others, in order to assess which aspects of the dental environment could be positive or negative for the successful cooperation of the patient with autism. The data from the parent questionnaires were subsequently analyzed and used during the desensitization of individual children. The questionnaire was made by the dentist with the parents and with one of the special needs center psychologists. This was important because it helped to identify the procedures carried out by the education center in order to facilitate the children’s access to dental treatment.

### 2.4. Examination

Prior to the dental examination, all of the participating children completed a systematic desensitization procedure within the general program of the Special Education Center. Desensitization consisted of a pre-examination to familiarize the child with the dental office, the techniques, the staff and the instruments that would be used during the child’s visit.

**Figure 1:**
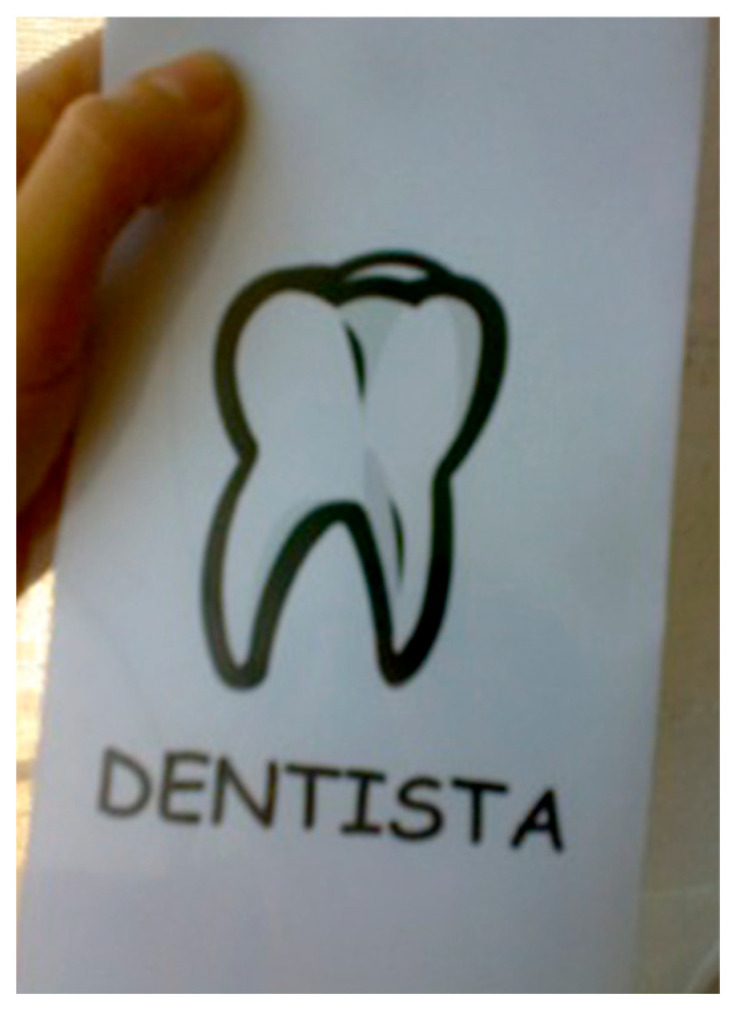
Representative visual cues of the dental procedure.

Representative visual cues of the dental procedure were created. In our study, we used a drawing of a molar as the actual examination key (Figure 1).

Along with the presentation of this pictogram, a plastic dental mirror was shown to the child so it could be related to the activity coming next. Later, information was gradually given to the children prior to the actual visit. Each child was given information at different times, which were selected by the teachers and caregivers at the special needs center who knew the children.

During the desensitization period, photo albums containing photographs with everything to do with the procedure were presented to the children.

After viewing the photo album, video modeling was used. Children watched a video of a girl undergoing a dental examination, which was a simulation of what would occur during the dental visit. The dentist was the same dentist that was going to perform the examination of the patients.

Finally, training was carried out with the children. A dental visit was simulated in a classroom at the center. There was a waiting room, and the office had a desk chair representing the dental chair as well as a light and a little table, to ensure it was close to reality. The children’s teachers acted as dentists in this first phase, wearing white coats and gloves and using mirrors and plastic probes.

Each group underwent the same entire sequence twice before going to the real dental office. The latency period between the last desensitization training and the real situation was one day for the first group and seven days for the second group.

On the actual dental examination day, the children were informed of the activity in advance by their teachers using the visual cues and the dental mirror.

Children went to the Department of Dentistry to undergo a dental examination in their designated groups. The same dentists that visited the special needs center were the ones that performed the examinations in the presence of the teachers or parents, as per the child’s preference, to reduce anxiety.

The presence of the children’s teachers, who had participated in the desensitization process, was suggested for a positive effect on the children´s overall experience.

Several steps were planned within the examination procedure. Thus, every step could be assessed upon completion, even if the examination had not been completed. These steps are shown in Table 1.

It was agreed that a step was to be considered fulfilled despite additional verbal or physical help needed from other sources such as the dentists, the psychologist that worked in the center or the educators. These data were also recorded and analyzed later to assess the impact of help needed on patient cooperation.

The visits to the Department of Dentistry were video recorded by a member of the research team in order to analyze the results obtained in every case.

Two data cards were created to evaluate the steps reached by every child:

- Data card recording of the dental experience portrayed in the video.
- Data card recording of the achievement of steps in the dental examination.

Two people (one from the education center and another from the Department of Dentistry) watched the video independently. Once the data were recorded, the inter-rater reliability was measured and tested. In order to achieve this, the percent agreement was used for two alternative categories, and the Kappa Index of Agreement was used for dichotomic categories. The reliability results ranged from 0.71 to 1.0 in the Kappa Index (very high reliability) and from 69% to 100% regarding the percentage of total agreement. If there was any disagreement, a third person was brought in to solve the disagreement.

### 2.5. Statistical Analyses

The data were analyzed using SPSS (Version 17.0) (SPSS Inc., Chicago, IL, USA). Descriptive statistics of the data obtained from the questionnaire were provided for an overview of the findings.

The dependent variable was the number of steps achieved in the examination protocol. The independent variables were characteristics of the sample group, such as the age, and the latency time between desensitization and the dental exam.

The number of patients was low, and we considered that the distribution was not normally distributed, so a non-parametric test was used, the Mann–Whitney and Kolmogorov Smirnov tests, and an ANOVA distribution with an H of Kruskal Wallis test.

A *p* value of 0.05 was considered statistically significant.

## 3. Results

### 3.1. Questionnaire Results

A total of 82% (*n* = 15) of the children suffered from hyperactivity disorder, which prevented them from being quiet for any extended period of time (which therefore makes dental visits even more difficult).

More than 50% (*n* = 10) of the parents felt that the sound of aspiration or other loud noises near the children had a negative effect. Furthermore, 35% (*n* = 7) of the sampled children could not tolerate direct light on their faces.

A total of 94% (*n* = 18) of the children liked feeling human contact on their faces. This was advantageous because certain manipulations and contact with the face were implicit in an examination.

Regarding the questions related to the children’s previous dental experiences, we found that 83% (*n* = 16) had never been to a dental office. In the remaining group, dental treatment that was needed in emergency situations in each of these cases was considered by parents to be a minimally traumatic experience.

A total of 47% (*n* = 9) of the children experienced involuntary movements, during which they could potentially injure themselves.

### 3.2. Examination Protocol Results

Table 2 summarizes the results regarding the completion of steps in the dental examination, including the number and percent of children who achieved each step.

For the established age groups, no statistically significant relationship was found to be related to the highest step achieved.

The latency time was analyzed in relation to the highest step of the protocol reached.

**Figure 2:**
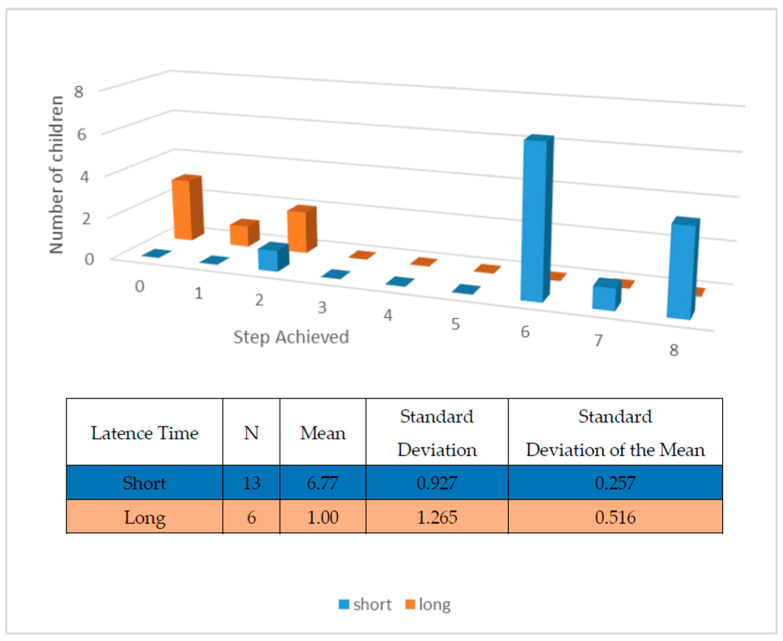
Number of children who completed the different steps in the examination protocol in relation to the latency time between the information and the examination (*p* < 0.01). The short latency time is 1 day prior to examination and the long latency time is 7 days.

Figure 2 shows the steps completed by each child related to the time elapsed between the information given in advance and the actual dental examination. There was a remarkable difference between the children who received the information just before the examination date and the children who experienced a longer latency period between receiving the information and experiencing the examination. The mean latency time for the short time delay was 1 day, and the mean time for the long time delay was 6.77 days because not all of the children were given the information 7 days before the examination.

## 4. Discussion

It has been detailed by certain authors that some stimuli can affect the behavior of autistic children during new experiences, such as noises or smells [12,17,18,27,28]. In our study, we found that noise could be a negative stimulus of the children’s behavior, as informed by the parents in the questionnaire.

As shown in Table 2 of the results, most of the children in the sample (with percentages ranging between 68% and 90% of group) completed all steps of the dental examination except the last step (presumably the one causing the greatest anxiety).

Other variables could have carried much more weight for determining the success in achieving more steps of the protocol, such as the support received and the promoting and teaching skills at the education center. We believe these should be part of future research.

The study variable was the latency time between the information provided in advance (completing the desensitization sequence in the Special Education Center) and the real examination.

We have found no references to this factor in other studies in this regard [17,18,21,25,28], but other authors [29] studying video modeling have reported that the information should be provided just before the actual situation, stressing the importance of this short time delay.

A report by Bäckman and Pielbro [25] and other studies of people with disabilities [21,27,28] have illustrated the importance of providing the information in advance, although latency periods are not referred to. Moreover, according to the educators at the Special Education Center, due to the characteristics of autistic children, this information must be visual and should be presented by means of a photo album or similar resources. According to the results obtained in our study, the provision of this information must take place as close to the visit as possible and must represent the real situation as accurately as possible.

The presence of some of the educators participating in the desensitization procedure is one of the essential factors in the development of the real situation. Educators are better able to provide verbal support.

Some authors [12,17,18,27,30] agree on the benefits of the presence of parents or educators who have cooperated in previous desensitization procedures.

Bäckman and Pielbro [25] also included the participation of parents or teachers to show the dental material to the child and to offer the relevant information in advance.

Other authors [12,17,27,30] also agree on the need to use more than one of the established techniques for behavior management in order to achieve the established aims in a real dental situation. The non-pharmacological techniques used by these authors and in our study, such as desensitization or video modeling, are good behavior management tools. Agreeing with authors such as Lewis, we can say that there is no “one size fits all” approach to dental and oral care for children with autism spectrum disorders [18].

As a result of our cooperation with a Special Education Center, we developed a procedure for dental treatment within the habitual activity of the center, following the established criteria of their programs for the development and adaptation of the abilities of the center´s pupils. This procedure is based on the systematic desensitization and latency time of our study. This is still under development with new tools such as reality goggles and tablets, which could help the children during the examination steps.

When evaluating the results obtained here, it is necessary to take into account that the children were evaluated after receiving the information in advance, which is essential for proper planning and the regulation of their behavior.

In terms of the strengths and limitations of this research, caution should be taken when considering statistical results because not all children could complete the study in the same way; some of them needed varying degrees of verbal or physical support. As a result of their different limitations, the children could not be homogeneously grouped nor independently studied. This does not reduce the importance of the results from the point of view of the research team; we believe that all the findings derived from this project must be interpreted within the context that they were developed.

We believe, in accordance with the teachers and caregivers of the special needs center and the parents of the children, that new technological advances could help the autistic population with dentist appointments as well as in other areas of their lives. We are also working with other lines of research such as virtual reality.

## 5. Conclusions

There is no “one size fits all” approach to dental and oral care for children with autism spectrum disorders.

It is important to emphasize the importance of providing information, such as visual cues, in advance when dealing with autistic patients. We found that this information must be as accurate as possible in portraying what will actually happen in the dental office. The shorter latency time provided the best results in our study.

It is also important to develop a simulated clinic. In the present investigation, we observed that the simulation should replicate the real situation as closely as possible.

During clinical procedures, behavior management techniques implemented in dentistry as a reinforcement of previous desensitization techniques should be taken into account.

The presence of parents, educators, and other key people in the autistic child’s social environment is desirable.

Although we were satisfied with the results of this study, we agree that more research is needed regarding the dental treatment of autistic children.

## Figures and Tables

**Table 1:** Steps in the examination protocol.

Steps Planned in the Examination Protocol
1. Sits in dental chair: seated in dental chair with legs extended but without lying back in the chair.
2. Lies in dental chair: leaning with legs extended.
3. Tolerates direct light on face.
4. Opens mouth.
5. Tolerates physical examination of the face: lets dentist manipulate the exterior of the mouth.
6. Tolerates physical manipulation of the interior of the mouth.
7. Opens mouth in front of a mirror.
8. Tolerates examination of the interior of the mouth with the mirror, keeping the mouth wide open.

**Table 2:** Percentage of children who completed the different steps of the protocol during the real dental examination.

Steps of the Dental Examination Protocol *n*: 19	Achieved without Help *n* (%)	Achieved with Help *n* (%)	Total Achieved *n* (%)	Not Carried out *n* (%)
1. Sits down on dental chair	6 (31.58%)	10 (52.63%)	16 (84.21%)	3 (15.79%)
2. Leans back in dental chair	6 (31.58%)	9 (47.37%)	15 (78.95%)	4 (21.05%)
3. Tolerates direct light on face	6 (31.58%)	8 (42.11%)	14 (73.69%)	5 (26.31%)
4. Opens mouth	6 (31.58%)	7 (36.84%)	13 (68.42%)	6 (31.58%)
5. Tolerates physical examination of the face	6 (31.58%)	7 (36.84%)	13 (68.42%)	6 (31.58%)
6. Tolerates physical manipulation of the mouth	6 (31.58%)	7 (36.4%)	13 (68.42%)	6 (31.58%)
7. Opens mouth in front of a mirror	3 (15.79%)	3 (15.79%)	6 (31.58%)	13 (68.42%)
8. Tolerates examination of the interior of mouth, keeping it wide open	3 (15.79%)	1 (5.26%)	4 (21.05%)	15 (78.95%)

## Data Availability

Data are available upon reasonable request.

## Supplementary Materials

The following supporting information can be downloaded at: https://www.mdpi.com/article/10.3390/children10050796/s1, File S1: Parent questionnaire (Spanish).
