# Supplementary material for: Importance of Desensitization for Autistic Children in Dental Practice
Source: Children (Basel). 2023 Apr 28;10(5):796. doi: 10.3390/children10050796 (PMC10217695; doi:10.3390/children10050796)
Supplement: Supplementary file 1 [file children-10-00796-s001.zip › children-2154544-supplementary.pdf]

DEMOGRAPHIC DATA

Data of the person responsible for the minor:

-Name and surname:..

-Parenthood :..

-Contact phone number :..

-Zip Code: ....

Date of birth patient: .... Current age (a/m):............... Sex: Male Female

Place of birth:..

What is the name you are called by at home: ....
Illness or Syndrome you suffer from: ....

Reason for inquiry: ....

MEDICAL HISTORY

Does your child have any allergies (medications, latex, food, etc.) Yes No List the patient's allergies below:

1. ....
2. ....
3. ....
4. ....

2. Have you had any diagnostic tests performed? Yes No

Do the parents suffer from any illness?

No Yes Which one?..

Do you have any siblings? No Yes How many?...................................Sex .... Age...

5. Does any family member have the same or a similar disease to the patient:

No Yes Who? ....

1. ....
3...

2. ....
4...

6. Did your child present any illness at the time of birth?

No Yes Which one?...

7. Does your son/daughter currently have any acute illnesses?

No Yes Which one?...

Does your son/daughter currently have any treatment for acute illness?

No Yes Which one? ....

Does your son/daughter suffer from any medical disorder or illness associated with his/her main illness: (hematological alterations, convulsions, digestive problems, sensory affectations)?)?

....

....

....

....

10. Do you currently have any treatment for these associated problems?

No Yes Which one? ....

Does your child have any respiratory or ear problems (ENT pathology)?

No Yes Which one? ....

12. Is your son/daughter undergoing or has your son/daughter undergone rehabilitation?

No Yes Which one? ....

BACKGROUND

13. Did the mother have any problems during pregnancy?

Yes No What type? ....

14. Was the delivery normal or did it present any problems?

Yes No What type? ....

15. Was your son/daughter born "full term" (9 months pregnant)?

Yes No How long? ....

16. Did your son/daughter require an incubator stay?

Yes No For how long? ....

17. Did your son/daughter require assisted ventilation?

Yes No For how long? ....

Did your son/daughter require transfusions?

Yes No How long? ....

19. Was the delivery natural? yes no

If not, indicate: Cesarean section Forceps

20. Did your son/daughter have any oxygen deprivation problems in the period immediately after his/her birth?

Yes No How long? ....

21. How much did your son/daughter weigh at birth?...

22. What was the size of your son/daughter at birth? ....

23. What type of breastfeeding did your child have?

Maternal For how long? ....

Artificial How long? ....

24. Does your child use a pacifier?

No Yes from.....................to.........................

Did you dip it in any substance (sugar, honey, etc)?

Yes No Which one?...

25. Did you use a bottle in feeding your son/daughter?

No Yes from.....................to.........................

26. Do you add any type of sweetener (any substance that serves to sweeten) to your child's food, bottle or teat?

No Yes Which one? ....

HABITS

27. Does your son/daughter have or has he/she had the habit of thumb sucking?

Yes No from.................. to..................

For how many hours?......................... Which finger?..............................

Not the finger, but other objects; pens, pencils, sheets ....

....

28. Does your child bite his/her nails?

No Yes from.................. to..................

Does your child's teeth grind?

No Yes from.................. to..................

30. Does your son/daughter have any other habits?
....

No Yes from.................. to..................

Which one?..

31. Does your child snore during sleep?

No Yes Do you have apnea episodes? Yes / No

32. Have you used or did you use any measure to correct these habits?

No Yes Which one?...

With what result?
....

DENTAL HISTORY

Do you know your child's current oral health status?

NoYesWhy ? ....

34. Have you ever been examined by a dentist before?

No Yes Date:..

35. Was your first visit to the dentist pleasant?

Yes No For what reason?...

36. How often do you go to the dentist?

1 time every 6 months 1 time per year

Once every 2 years more than 2 years

37. What was your son/daughter's first visit?

For review For pain

38. Do you have a toothbrush at home? No

Yes

39. Do you have a toothbrush at school? No

Yes

Is it electric?

No Yes

Is it electric?

No Yes

40. Do you brush daily? No Yes

41. How many times a day are your son/daughter's teeth brushed?

None one more than one how many ....

42. At what time of the day do you brush your teeth?

After meals When you remember

43. How much time is spent brushing? ....

44. How often do you change your child's toothbrush? Every month Every 2 months

Every 3 months Above 6 months

45. Does he/she brush his/her teeth alone? No Yes Sometimes

46. Do you brush your child's teeth?

No Yes Sometimes

47. Do you use toothpaste in your brushing?

No YesWhich ? ....

48. How much toothpaste do you use?

Nothing One pellet Slightly less than half of the brush Slightly more than half of the brush Whole brush

49. Do you use dental floss or fluoride rinses?

Dental Floss Fluoride rinses Both

Guideline used..

50. In successive visits, has your son/daughter been treated?

No Yes Don't know

Date: ....Type of treatment: ....

51. Have you suffered any blows to your teeth?

No Yes How old were you? ....

Dentition: Temporary (deciduous) Permanent (adult)

DIET AND NUTRITION

52. What type of nutrition is provided to your child?

Oral Parenteral From.................... to.......................

53. Number of meals per day ....

54. Do you eat anything between meals?

NoYesWhat is it usually? ....

55. As a reward, do you receive sweets, candies...?

NoYesWhat is it usually? ....

56. What is your child's favorite type of food?

SweetYes No

SaltyYes No

ColdYes No

HotYes No

CreamyYes No

CrunchyYes No

Others

....

Does your son/daughter ingest inedible substances?

No Yes

Which ones?..

58. Does your son/daughter have difficulty swallowing food? Why?

....

No Yes

59. Does your son/daughter take a soft diet?

Not Always Usually

60. Does your son/daughter have frequent regurgitation or vomiting after meals? No Yes No

Thank you for your invaluable help in answering this questionnaire about your son/daughter. The answers you have provided here are completely confidential, and will only be used by the research team to develop a specific treatment and prevention plan for each child.
